# Modulation of Stop Codon Read-Through Efficiency and Its Effect on the Replication of Murine Leukemia Virus

**DOI:** 10.1128/JVI.00898-14

**Published:** 2014-09

**Authors:** Eszter Csibra, Ian Brierley, Nerea Irigoyen

**Affiliations:** Division of Virology, Department of Pathology, University of Cambridge, Cambridge, United Kingdom

## Abstract

Translational readthrough—suppression of termination at a stop codon—is exploited in the replication cycles of several viruses and represents a potential target for antiviral intervention. In the gammaretroviruses, typified by Moloney murine leukemia virus (MuLV), *gag* and *pol* are in the same reading frame, separated by a UAG stop codon, and termination codon readthrough is required for expression of the viral Gag-Pol fusion protein. Here, we investigated the effect on MuLV replication of modulating readthrough efficiency. We began by manipulating the readthrough signal in the context of an infectious viral clone to generate a series of MuLV variants in which readthrough was stimulated or reduced. In carefully controlled infectivity assays, it was found that reducing the MuLV readthrough efficiency only 4-fold led to a marked defect and that a 10-fold reduction essentially abolished replication. However, up to an ∼8.5-fold stimulation of readthrough (up to 60% readthrough) was well tolerated by the virus. These high levels of readthrough were achieved using a two-plasmid system, with Gag and Gag-Pol expressed from separate infectious clones. We also modulated readthrough by silencing expression of eukaryotic release factors 1 and 3 (eRF1 and eRF3) or by introducing aminoglycosides into the cells. The data obtained indicate that gammaretroviruses tolerate a substantial excess of viral Gag-Pol synthesis but are very sensitive to a reduction in levels of this polyprotein. Thus, as is also the case for ribosomal frameshifting, antiviral therapies targeting readthrough with inhibitory agents are likely to be the most beneficial.

**IMPORTANCE** Many pathogenic RNA viruses and retroviruses use ribosomal frameshifting or stop codon readthrough to regulate expression of their replicase enzymes. These translational “recoding” processes are potential targets for antiviral intervention, but we have only a limited understanding of the consequences to virus replication of modulating the efficiency of recoding, particularly for those viruses employing readthrough. In this paper, we describe the first systematic analysis of the effect of increasing or decreasing readthrough efficiency on virus replication using the gammaretrovirus MuLV as a model system. We find unexpectedly that MuLV replication is only slightly inhibited by substantial increases in readthrough frequency, but as with other viruses that use recoding strategies, replication is quite sensitive to even modest reductions. These studies provide insights into both the readthrough process and MuLV replication and have implications for the selection of antivirals against gammaretroviruses.

## INTRODUCTION

**FIG 1:**
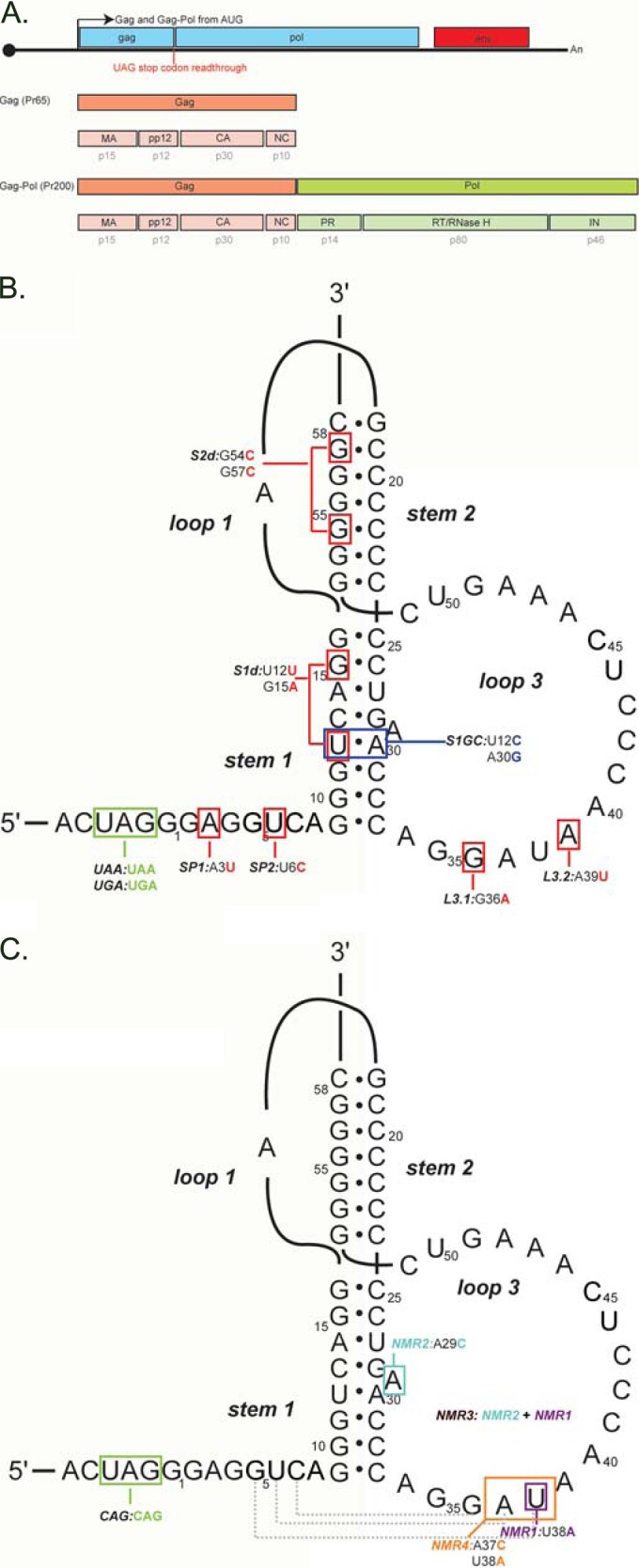
MuLV genomic RNA and secondary structure of the readthrough signal. (A) The 5′ end of the MuLV gRNA encodes polyproteins Gag (Pr65) and Gag-Pol (Pr200), separated by a UAG codon that is subject to stop codon readthrough. Upon dimerization of the protease domain after budding, the Gag and Gag-Pol polyproteins are processed into smaller polypeptides, as illustrated. (B) The readthrough signal is located immediately 3′ of the *gag* UAG codon and consists of a compact hairpin-type pseudoknot consisting of two stems, S1 and S2, connected by a single base, loop L1, and an 18-nucleotide loop, L3. In this model (9), the 8-nucleotide spacer between the UAG stop codon (boxed in green) and pseudoknot is unstructured (9). The readthrough signal is labeled with the first-generation mutations and color coded to indicate the predicted effect on pseudoknot stability (blue, stabilization; red, destabilization). Mutations of the stop codon are shown in green. (C) The MuLV readthrough signal, with additional base pairing as proposed by Houck-Loomis and colleagues (10), is shown as dashed lines, annotated with the second-generation mutations. In green is the 100% readthrough mutation (CAG) used for the two-plasmid system.

Almost all retroviruses employ programmed ribosomal frameshifting or stop codon readthrough as a means to express their replicase enzymes (Pol, including reverse transcriptase [RT]) as a C-terminal extension of the polyprotein of structural proteins (Gag). Frameshifting and readthrough are examples of translational recoding signals that suspend the normal readout of the genetic code and promote alternative translation strategies (1–4). In gammaretroviruses, typified by murine leukemia virus (MuLV), *gag* and *pol* are in the same reading frame, separated by a UAG stop codon. Some 5 to 10% of ribosomes translating *gag* read through the stop codon, inserting glutamine, and continue translation to produce the Gag-Pol polyprotein (5). In MuLV, a compact RNA structure located downstream of the *gag* stop codon has been shown to direct the recoding process (see Fig. 1) (6–10). Nuclear magnetic resonance (NMR) studies have revealed an active pseudoknot conformation, which represents a minor (6%) component that exists in equilibrium with an inactive conformation (10). How the active structure stimulates readthrough is not clear, but it could involve direct modulation of ribosome function (11, 12), interference with release factor activity through steric hindrance, sequestration or modulation of other proteins involved in termination, or recruitment of other factors that modulate release factor function (4, 13).

Strikingly, whether employing frameshifting or readthrough, retroviruses express Gag-Pol at a level of 5 to 10% of that of Gag, and this is thought to underlie the ratio of structural to nonstructural proteins that get assembled into virus particles (14). There is interest in understanding the biological relevance of maintaining the Gag-Pol/Gag ratio as it represents a potential target for antiviral intervention (15–17). It is well established that viruses expressing only Gag are essentially inviable (18–21), and several groups of investigators have found that viruses expressing only Gag-Pol are also severely disabled, perhaps because the overabundance of Gag-Pol causes premature activation of the viral protease and virion assembly defects (19, 20, 22–24). Previous work has revealed that HIV-1 replication is acutely sensitive to minor (2-fold) increases or decreases in frameshifting efficiencies (14, 25–27), and this is supported by studies of sequence variation between isolates (28, 29); yet in Rous sarcoma virus (RSV), up to an 8-fold reduction or a 3-fold increase in frameshifting is tolerated before viral propagation is disrupted (24). A limitation of many of the studies to date, however, is that a broad range of recoding efficiencies has not been tested, particularly virus variants with elevated recoding efficiencies. Furthermore, several of the data sets are complicated by secondary effects on protein expression or function resulting from the introduction of nonsynonymous base substitutions within recoding sites (e.g., protease function in RSV [24]), or changes that affect overlapping coding sequences (e.g., p6* of HIV-1 [30]).

In this study, we investigated the effect on MuLV replication of modulating readthrough efficiency. We first manipulated the readthrough signal in the context of an infectious viral clone to generate a series of MuLV variants in which readthrough was stimulated or reduced. In carefully controlled infectivity assays, judged by the 50% tissue culture infective dose (TCID_50_), protein expression, and reverse transcriptase activities, it was found that reducing the MuLV readthrough efficiency (RTE) 4-fold led to a marked defect and that a 10-fold reduction essentially abolished replication. However, a 2-fold stimulation had very little effect on replication, and, indeed, up to an ∼8.5-fold stimulation of readthrough (equivalent to up to 60% readthrough) was well tolerated. These high levels of readthrough were achieved using a two-plasmid system, with Gag and Gag-Pol expressed from separate infectious clones. We also modulated readthrough efficiencies by silencing expression of eukaryotic release factors 1 and 3 (eRF1 and eRF3) or by introducing aminoglycosides into the cells. The data obtained reveal that gammaretroviruses tolerate a substantial excess of viral Gag-Pol synthesis but are very sensitive to a reduction in levels of this polyprotein.

## MATERIALS AND METHODS

### MuLV proviral clone.

The full-length MuLV Moloney strain proviral clone pNCA (31) (GenBank accession number AF033811.1) was a kind gift of S. P. Goff, Columbia University.

### Plasmids.

Site-directed mutagenesis of the MuLV readthrough signal was carried out in plasmid pING14.2/XhoI, a variant of pING14.2 (32) containing a unique XhoI site in the polylinker region. A 2,709-bp XhoI/SalI fragment from pNCA, encompassing the MuLV *gag-pol* overlap region (genomic coordinates 2009 to 4718), was cloned into SalI- and XhoI-digested pING14.2/XhoI and subjected to PCR mutagenesis. Subsequently, 1,121-bp BclI/XhoI fragments from this plasmid were inserted into BclI/XhoI-digested pNCA to generate the pNCA series of virus mutants. The same strategy was used to generate a virus lacking protease activity (substitution of Asp for Asn at the PR active site) and truncated in Pol (introduction of an optimal premature stop codon, UAAA [underlined]). This virus (Pr− TAAA) expresses an unprocessed Gag-Pol polyprotein of 90 kDa. For assessment of readthrough efficiencies in transfected tissue culture cells, the dual-luciferase readthrough reporter vector pDluc was employed (a kind gift of M. Howard, University of Utah) (33). DNA fragments of 100 bp spanning the MuLV readthrough region and flanked by XhoI and BglII restriction sites were derived by PCR amplification and ligated into appropriately cleaved pDluc vector. All sequences were confirmed by dideoxy sequencing.

### Readthrough assays in tissue culture cells.

Cells were maintained in Dulbecco's modified Eagle's medium supplemented with 10% (vol/vol) fetal calf serum (DMEM-FCS). Cells were seeded in dishes of a 24-well plate and grown for 16 h until 80% confluence was reached. Plasmids were transfected using a commercial liposome method (TransIT-LT1; Mirus). Transfection mixtures (containing plasmid DNA, serum-free medium [Opti-MEM; Gibco-BRL], and liposomes) were set up as recommended by the manufacturer and added dropwise to the tissue culture cell growth medium. The cells were harvested at 24 h posttransfection (hpt), and reporter gene expression was determined using a dual-luciferase assay system kit (Promega). Values for readthrough efficiency were calculated by dividing the expression ratio of firefly luciferase to Renilla luciferase (Fluc/Rluc) of the samples by the Fluc/Rluc ratio of the in-frame control. Statistical analysis was performed using methods described by Jacobs and Dinman (34).

### Virus assays.

MuLV replication was assessed by transfection of proviral clone constructs and subsequent infection of Rat2 cells with virions released from transfected cells. 293T monolayers at 40% confluence were prepared in six-well plates and transfected with pNCA or a mutant derivative. To assess virus production efficiency, cells were harvested at 72 hpt and subjected to SDS-PAGE and immunoblotting for viral proteins. A cellular protein (glyceraldehyde-3-phosphate dehydrogenase [GAPDH]) was used as a loading control. Tissue culture supernatants were filtered through a 0.45-μm-pore-size filter prior to infection of fresh monolayers or biochemical analysis. Filtered supernatant (500 μl) from transfected cells was added directly to six-well plates of Rat2 monolayers at 10% confluence and incubated for 1 h at 37°C, and the volume was adjusted to 3 ml with fresh medium (DMEM with 2% FCS) in the presence of 30 μg/μl of DEAE-dextran hydrochloride (Sigma). Cells were incubated at 37°C and 10% CO_2_ for 4 days. Cells were harvested directly in Laemmli's sample buffer, and proteins were analyzed by immunoblotting. Viral supernatant was sterile filtered and used for protein and RNA analysis.

### Immunoblotting.

Proteins were separated by 10% SDS-PAGE and transferred to nitrocellulose membranes. These were blocked for 30 to 60 min with 5% powdered milk (Marvel) in PBST (137 mM NaCl, 2.7 mM KCl, 10 mM Na_2_HPO_4_, 1.5 mM KH_2_PO_4_, pH 6.7, and 0.1% Tween 20) and probed with polyclonal rabbit anti-MuLV p30 (ab130757; Abcam) (1:2,000 in Marvel-PBST) and monoclonal mouse anti-GAPDH (G8795; Sigma-Aldrich) (1:20,000 in Marvel-PBST). Membranes were incubated in the dark with an IRDye-conjugated secondary antibody in PBST (IRDye 800CW donkey anti-rabbit IgG_H+L_ and IRDye 680RD goat anti-mouse IgM [μ chain specific]). Blots were scanned, and bands were quantified using an Odyssey infrared imaging system (Li-Cor).

### Dot blot assays.

Filtered supernatants from transfections were serially diluted 2-fold and applied to a phosphate-buffered saline (PBS)-washed nitrocellulose filter in duplicate using a Minifold Micro-Sample Filtration Manifold (Schleicher & Schuell). Samples were allowed to bind; they were washed twice with PBST and blocked, probed, and quantified as described above.

### Reverse transcriptase assays.

Filtered supernatants from transfections were serially diluted 2-fold in DMEM containing 10% FCS. Supernatant (5 μl) was mixed with 20 μl of gammaretroviral RT assay buffer [50 mM Tris (pH 8.3), 0.6 mM MnCl_2_, 60 mM NaCl, 20 mM dithiothreitol (DTT), 0.05% (vol/vol) NP-40, 10 μg/ml poly(A), 5 μg/ml oligo(dT), 10 μM dTTP, 8 μCi of [α-^32^P]dTTP] and incubated for 2 h at 37°C. Reaction mixture aliquots (5 μl, in triplicate) were pipetted onto DE81 paper in grid formation and allowed to dry. The DE81 paper was washed in 0.5 M sodium phosphate buffer (four times for 5 min each), water (two times for 1 min each) and 100% ethanol (1 min) and allowed to dry completely before exposure to X-ray film. Incorporation of [α-^32^P]dTTP was quantified by phosphorimaging.

### TCID_50_ assays.

Virus replication was assessed using a 50% tissue culture infective dose (TCID_50_) assay. One day prior to infection, Rat2 cells were seeded in 96-well plates at 4 × 10^3^ cells/well in a final volume of 100 μl/well. Sterile-filtered supernatant was harvested at 72 hpt from a six-well plate transfected with a pNCA derivative plasmid, and the supernatant was serially diluted 10-fold in DMEM (supplemented with 2% FCS and 30 μg/ml DEAE-dextran hydrochloride). An aliquot of each diluted sample (100 μl/well) was added to each of five wells of a 96-well plate containing Rat2 cells seeded as above. At 3 days postinfection (dpi), the culture medium was replaced with 100 μl of Opti-MEM, and the cells were stained 12 h later. For staining, the cells were washed with PBS and fixed/permeabilized in methanol-acetone (1:1) for 30 min. After two further washes with PBS, nonspecific binding sites were blocked by incubation in 3% bovine serum albumin (BSA) in PBST for 30 min. Cells were probed with anti-MuLV p30 antibody (1:2,000 in BSA-PBST; 50 μl for 1 h at room temperature), washed, and incubated with a fluorescent secondary antibody (Alexa Fluor 568 goat anti-rabbit IgG [A-11011; Invitrogen]; 1:1,000 in PBST, 1 h at room temperature). Staining was visualized using an Olympus IX70 fluorescence microscope. Wells containing any brightly staining cells were scored as infected. Experiments were conducted using duplicate biological repeats, each diluted in parallel and used to infect five rows of wells. For each biological repeat, the 50% endpoint titer was calculated according to the method of Reed and Muench (35).

### Generation of silenced Rat2 cells.

eRF1 and eRF3 short hairpin (sh) oligonucleotides with 19-nucleotide-long targeting sequences were cloned in pRETRO-SUPER (Netherlands Cancer Institute/Antonie van Leeuwenhoek Hospital), previously digested with BglII and HindIII, to yield pRETRO-SUPER-eRF1 and pRETRO-SUPER-eRF3. These plasmids, and an empty plasmid as a control, were transfected using TransIT-LT1 transfection reagent (Mirus) into packaging cell line 293T, with pCL-anfo as a helper plasmid. pRETRO-SUPER expresses a transcript containing the viral packaging signal, the H1-short hairpin RNA (shRNA) cassette, and the puromycin resistance gene. At 48 h posttransfection, supernatant was collected, filtered (0.45-μm pore size), and used to infect Rat2 cells three times over a 24-h period, with addition of Polybrene (at 4 μg/ml). Transduced cells were selected and cultured in the presence of puromycin (at 2 μg/ml).

### Isolation and sequence of RNA from released virions.

Culture medium (1 ml) was sterile filtered and incubated for 30 min at 37°C with 5 μl of Turbo DNase I (Ambion), followed by the inactivation of the enzyme by the addition of 15 mM EDTA. The reaction mixture was centrifuged at 154,000 × *g* for 1 h at 4°C, and the pellet was gently resuspended in 100 μl of 50 mM Tris (pH 7.5), 10 mM EDTA, 1% SDS, 100 mM NaCl, and 100 μg of proteinase K and incubated for 30 min at 37°C, followed by phenol-chloroform extraction. Ten microliters of sample was reverse transcribed using avian myeloblastosis virus RT (Promega); the resulting cDNA was PCR amplified and cloned into the pGEM-T-Easy Vector (Promega) before individual clones were selected for sequencing.

## RESULTS

### Modulation of MuLV readthrough efficiency through site-specific mutagenesis.

**FIG 2:**
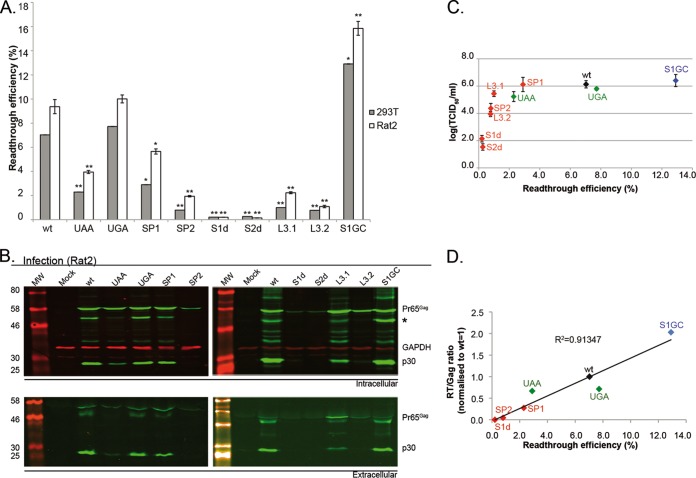
Readthrough efficiencies and virus replication analysis of first-generation readthrough signal mutants. (A) MuLV readthrough efficiencies in 293T and Rat2 cells. Cells were transfected with pDluc-MuLV or a first-generation mutant derivative (Fig. 1B), and 24 h later, lysates were prepared and assayed for Renilla and firefly luciferase. Data represent mean values from at least eight samples over three independent experiments. Error bars represent standard errors of the means (SE). Samples were subjected to two-tailed *t* tests if they corresponded to a normal distribution or to Mann Whitney U tests if not. *, *P* < 0.01; **, *P* < 0.001, compared to the WT. (B) 293T cells were transfected with pNCA or a first-generation mutant derivative, and released virus was used subsequently to infect Rat2 cells. Cell lysates (upper panel) and supernatant virus (lower panel) from Rat2-infected cells were analyzed by 10% SDS-PAGE and immunoblotting using a polyclonal anti-CA (p30) serum. Molecular weights (MW; in thousands) are indicated on the left. GAPDH was used as a loading control. All viral proteins were detected with a green fluorescent secondary antibody, and GAPDH was detected with a red fluorescent secondary antibody. An asterisk indicates a Pr65^Gag^ processing intermediate with a slightly raised intracellular level. (C) TCID_50_ assays were performed with serial dilutions of 293T-transfected cells as described in Materials and Methods. Samples were harvested at 96 hpi and stained for Gag, and plates were imaged and scored for the presence of fluorescent signal. TCID_50_ titers were calculated according to Reed and Muench (35) and are represented as log-transformed values for clarity. Values represent the mean of duplicate titrations from two independent experiments, each internally normalized to the WT titer. Error bars represent standard errors. (D) Gag-Pol/Gag ratios in supernatant viruses from 293T-transfected cells (72 hpt) was estimated as the ratio of reverse transcriptase activity (RT) to Gag protein concentration, as judged by dot blot quantification. RT assay data are the mean of triplicate values, and dot blot numbers are the mean of two independent experiments in duplicate. The WT ratio was set to 1, and other samples were normalized to the WT. Only representative mutants were selected.

To investigate the importance to MuLV replication of maintaining the natural Gag-Pol/Gag ratio, we began by introducing mutations into the stimulatory RNA structure that were predicted to increase or decrease the RTE based on previous studies (6–9). The mutations were synonymous and designed to destabilize stem 1 (S1d) or stem 2 (S2d) or were single base changes in the spacer (SP1 or SP2) or loop 3 (L3.1 or L3.2) (Fig. 1B). Three other mutants were prepared: the UAG stop codon was changed to UGA or UAA, and a synonymous stabilizing change was introduced into stem 1, with a U-A pair replaced by a C-G pair (S1GC). To confirm the effects of the various mutations on readthrough, they were introduced into a dual-luciferase readthrough reporter plasmid (pDluc) (33) containing a 100-bp fragment of the MuLV readthrough signal placed between Renilla and firefly reporter genes. In this plasmid, readthrough of the MuLV *gag* stop codon allows expression of the Fluc gene as a fusion with the upstream Rluc gene, analogous to the expression of Gag-Pol. Readthrough efficiencies were calculated from the ratios of Fluc activity (in relative light units [RLU]) versus Rluc activity for each mutant, relative to its control. Controls consisted of the mutant constructs containing a CAG codon (to measure 100% readthrough) instead of the UAG stop codon (36) (see Materials and Methods). The various mutants were transfected into 293T and Rat2 cell lines, and readthrough was measured using dual-luciferase assays. (Fig. 2A). The RTE of the wild-type (WT) signal was 7% in 293T cells and 9% in Rat2 cells, and the mutations mostly inhibited readthrough (down mutants). Two (UAA and SP1) led to a decrease of 1.7- to 3.1-fold, three (SP2, L3.1, and L3.2) led to decreases between 4- and 10-fold, and the stem destabilizations (S1d and S2d) were greatly inhibitory (27- to 65-fold down in both cell types). Changing the stop codon to UGA had essentially no effect on readthrough (as seen before) (7, 37, 38), and the stem 1 stabilizing mutation (S1GC) indeed stimulated readthrough (an up mutant with about a 2-fold increase; 13 to 16% depending on cell type), consistent with a role for pseudoknot stem 1 stability in recoding (39, 40).

### Effect of modulating readthrough efficiency on MuLV replication.

The effect of these mutations was then assayed in the context of an infectious MuLV clone (pNCA). Monolayers of 293T cells were transfected, and at 72 h posttransfection, virus was harvested, filtered, and used to inoculate fresh Rat2 cell monolayers at 10% density. Cells were incubated at 37°C for a total of 4 days. Cell lysates (intracellular) were subjected to SDS-PAGE and blotting for Pr65^Gag^ (Gag) using a polyclonal anti-p30 (capsid [CA]) antibody (which also detects Pr200^Gag-Pol^ [Gag-Pol]). Culture medium was also harvested and sterile filtered, and aliquots were analyzed by immunoblotting (extracellular). As shown in Fig. 2B, mutants pNCA-SP2, -S1d, -S2d, and -L3.2 were found to be severely attenuated, with little (SP2 and L3.2) or no (S1d and S2d) evidence of their replication. There was also a clear deficiency in replication of the viruses in which RTE was reduced between 3- and 8-fold in the dual-luciferase assays (pNCA-UAA and -L3.1). The up mutant (pNCA-S1GC), however, replicated as efficiently as the WT virus. The only noticeable difference was a slightly raised intracellular level of a Pr65^Gag^ processing intermediate (Fig. 2B, asterisk), probably arising as a consequence of the increased RTE and thus the increased levels of viral protease. To obtain a more quantitative assessment of replication capacity, we carried out TCID_50_ assays (Fig. 2C). From this analysis, it was possible to group the viruses into four categories: nonreplicating (pNCA-S1d and -S2d), with a TCID_50_ some 4 to 5 logs lower than that of the WT virus; poorly replicating (pNCA-SP2 and -L3.2), with the TCID_50_ reduced by 1 to 3 logs; moderately replicating (pNCA-UAA and -L3.1), with a reduction of under 1 log; and fully replicating (pNCA-SP1, -UGA, and -S1GC). Thus, consistent with the data above, a 2-fold reduction in RTE (SP1) has no discernible effect on replication, mild reductions have some effect, and more than an 8-fold reduction abolishes virus replication. We also addressed the question of whether modulating readthrough efficiencies altered the proportions of Gag and Gag-Pol in released virions. This was achieved by comparison of the ratio of virion RT activities (describing Gag-Pol) and Gag content as measured by quantitative immunodot blotting (Fig. 2D) (24). We found that RT levels increased as RTE increased, whereas Gag levels decreased by a small amount (data not shown). A strong correlation (*R*^2^ = 0.91) was observed between the resultant RT/Gag ratios and the RTE, suggesting that the readthrough process may be important for determining this ratio.

### Extending the readthrough gradient.

**FIG 3:**
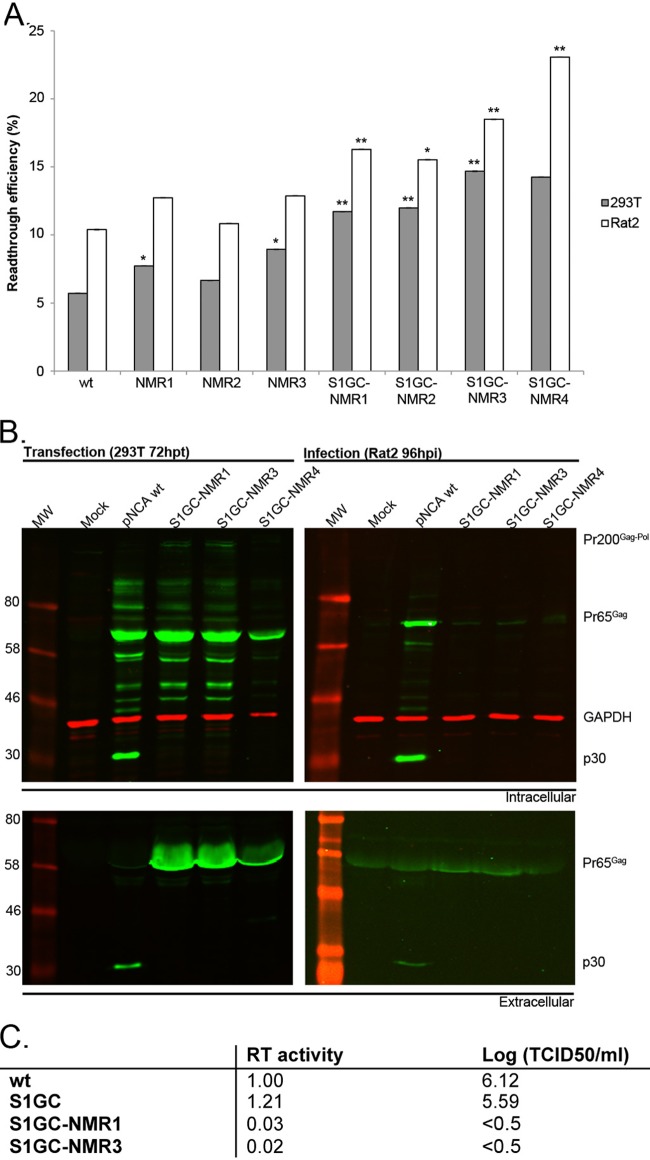
Readthrough efficiencies and virus replication analysis of second-generation readthrough signal mutants. (A) 293T and Rat2 cells were transfected with pDluc-MuLV or a second-generation mutant derivative (NMR1 to NMR4 in Fig. 1C) independently and in combination with the S1GC mutation. Readthrough efficiencies were calculated as described in the legend of Fig. 2A. Values represent the means of at least eight samples from three independent experiments, except for S1GC-NMR4, which was from one experiment. *, *P* < 0.05; **, *P* < 0.01, compared to the WT. (B) 293T cells were transfected with pNCA or second-generation mutant derivatives (left panel), and released virus was used subsequently to infect Rat2 cells (right panel). Cell lysates and supernatant virus were analyzed by 10% SDS-PAGE and immunoblotting using a polyclonal anti-CA (p30) serum. Molecular weights (MW; in thousands) are indicated on the left. GAPDH was used as a loading control. All viral proteins were detected with a green fluorescent secondary antibody, and GAPDH was detected with a red fluorescent secondary antibody. (C) RT assay results were the means of triplicate values and were normalized to WT (set to 1). TCID_50_ assays were performed with serial dilutions of supernatant of 293T-transfected cells as described previously, and titers are represented as log-transformed values. The value of <0.5 indicates that the titers were so low as to be undetectable; in our system, 0.5 is the minimum detectable titer. Values represent the means of two experiments.

To generate additional viruses with enhanced levels of readthrough, further mutations were introduced into the pseudoknot (second-generation mutants) (Fig. 1C) based on insights from NMR analysis (10). Two mutations, U38A and A29C (Fig. 1C, NMR1 and NMR2, respectively), were previously documented to increase the RTE from 8% to 20% in *in vitro* translation assays (10). These changes (NMR1 and NMR2) or their combination (NMR3), with or without the S1GC mutation (S1GC-NMRX, where X is 1 to 3), were introduced into pDluc-MuLV, and readthrough was measured by dual-luciferase assays in 293T and Rat2 cells (Fig. 3A). The experiment revealed that alone, the NMR1, NMR2, or NMR3 mutation led to only a slight increase of RTE compared to the WT in our system. However, combining these changes with the S1GC mutation led to marked stimulation (1.5- to 2.6-fold). The S1GC-NMR1 and S1GC-NMR3 mutations, exhibiting the highest RTEs, were introduced into the proviral clone, and replication was assessed as above. Both viruses exhibited dramatic phenotypes in virus assays (Fig. 3B). Cell lysates of transfected cells (Fig. 3B, top left panel), as well as virus-containing supernatants (bottom left panel), showed an accumulation of Gag and Gag-Pol polyproteins in comparison to the WT, whereas p30 was undetectable in these mutants. Released virions were noninfectious, judged by the almost complete absence of viral proteins in Rat2-infected cells at 96 hpi (Fig. 3B, right panel) and the baseline RT and TCID_50_ levels (Fig. 3C). As the U38A and A29C mutations (NMR1 and NMR3) were nonsynonymous, it seemed likely that the amino acid substitutions had interfered with protease activity. Indeed, the U38A mutation present in both of these viruses leads to an isoleucine-to-lysine change, introducing a positive charge and potentially disrupting interactions with targets or enzymatic activity. In an attempt to circumvent this problem, S1GC-NMR4 was prepared (Fig. 1C), which harbored all S1GC-NMR3 mutations plus an A37C substitution, changing the isoleucine to glutamine. Unfortunately, while this mutant exhibited the highest readthrough efficiency (14 to 23%,) (Fig. 3A), the virus phenotype was similarly defective (Fig. 3B), suggesting that the identity of this amino acid (isoleucine) may be essential to protease function.

### Nontargeted global deregulation of termination.

**FIG 4:**
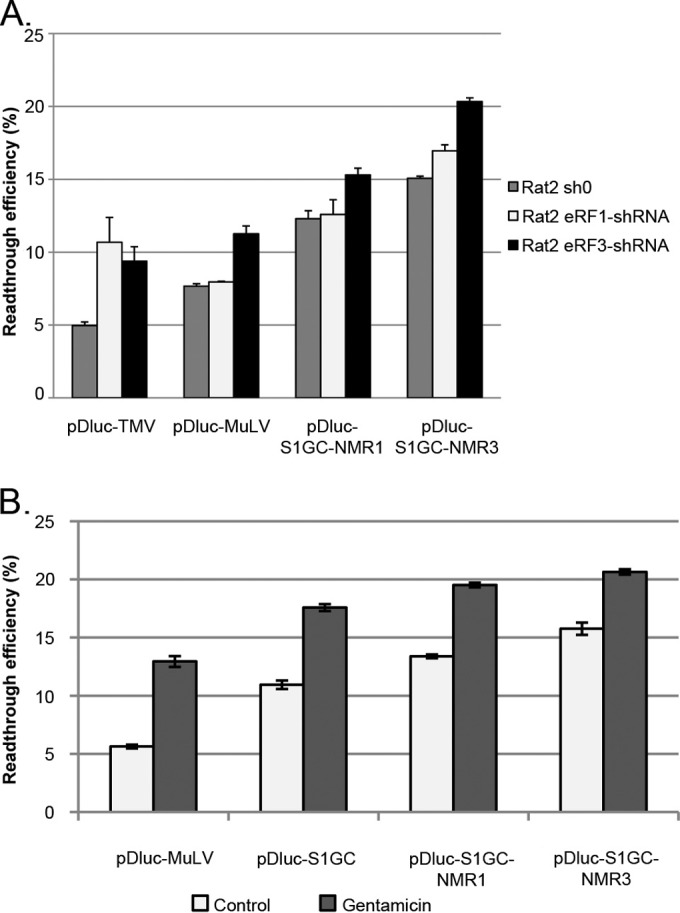
Nontargeted global deregulation of termination. (A) Effect of release factor knockdown on MuLV readthrough efficiency. Rat2 cells silenced for eRF1 (Rat2 eRF1-shRNA), eRF3 (Rat2 eRF3-shRNA), and a negative control (Rat2 sh0) were transfected with pDluc-TMV, pDluc-MuLV, pDluc-S1GC-NMR1, or pDluc-S1GC-NMR3, and 24 h later, lysates were prepared and assayed for Renilla and firefly luciferase. (B) Effect of gentamicin on MuLV readthrough efficiency. 293T cells were transfected with pDluc-MuLV, pDluc-S1GC, pDluc-S1GC-NMR1, and pDluc-S1GC-NMR3 in the absence or presence of gentamicin (750 μg/ml) and assayed for Renilla and firefly luciferase 24 h later.

As an alternative strategy to enhance readthrough levels, we sought to downregulate the efficiency of translation termination in the cell generally, first through depletion of eukaryotic release factors. eRF1 and eRF3 are responsible for recognizing stop codons in the ribosomal A site and, following GTP hydrolysis, begin the termination process of polypeptide release and ribosome disassembly (41–43). It is known that depletion of eRF1 enhances readthrough at all three stop codons as does the depletion of eRF3 in some cell lines (44). Consistent with this, it has been shown that MuLV RT binds to eRF1 to modulate suppression of translation termination, upregulating readthrough and creating a positive feedback loop that drives the synthesis of more *pol* products (45). Stable Rat2 cells with reduced eRF1 or eRF3 expression were produced by transduction with retroviruses expressing short hairpin RNAs (eRF1-shRNA and eRF3-shRNA). An empty retrovirus vector was used to generate a negative-control cell line (sh0). The levels of release factor expression were reduced by ∼65% for the eRF1 and ∼70% for the eRF3 cell lines (data not shown). Subsequently, cells were transfected with MuLV readthrough reporter plasmids (pDluc-MuLV, -S1GC-NMR1, and -S1GC-NMR3) or a positive-control vector (pDluc-TMV) containing the well-characterized readthrough signal of tobacco mosaic virus (TMV) (46) (Fig. 4A). TMV readthrough was stimulated 2-fold in both knockdown cell lines, consistent with the reduced levels of release factors. However, with the MuLV reporter constructs, RTE was stimulated in only the eRF3 knockdown cell line and by only a maximum of 1.5-fold. The lack of stimulation of MuLV readthrough in the eRF1 knockdown cell line, as opposed to the TMV signal (which comprises a UAG stop codon and a short 3′ stimulatory primary sequence context), was unexpected but may reflect a difference in the mechanism of readthrough manifest as differential sensitivity to eRF1 levels. Subsequently, we went on to compare MuLV replication in control and eRF3 knockdown cells in infectivity assays. Unfortunately, the virus replicated very poorly in the knockdown cells (data not shown), and it was impossible to say whether this was a consequence of the modest stimulation of RTE or the evidently poor growth rate of the knockdown cells.

An alternative approach to globally deregulate translation termination was the use of aminoglycosides which bind to 18S rRNA and interfere with small-subunit function by modulating A-site-decoding accuracy (47). This results in translational misreading at sense codons (misincorporation) and misreading of nonsense codons as sense codons (readthrough). An attractive reason for using aminoglycosides was the potential to titrate drug concentration to generate a range of RTEs for testing in virus viability assays. Gentamicin (48) was selected for this experiment, and dual-luciferase assays (data not shown) revealed that 750 μg/ml was the most effective concentration for stimulation of MuLV readthrough, increasing it by 1.5- to 2-fold and allowing a range of RTEs to be produced from 6% (pDluc MuLV no gentamicin) to 20% (pDluc-S1GC-NMR3 with gentamicin) (Fig. 4B). However, in virus infectivity assays, we found no effect of adding this optimal gentamicin concentration to cells following transfection of proviral clones [log(TCID_50_/ml) values for control versus gentamicin were 5.41 ± 0.09 and 5.44 ± 0.06, respectively]. Thus, the 1.5-fold increase in RTE that results from gentamicin treatment was insufficient to affect virus replication in a single-round assay.

### Modulation of effective MuLV readthrough efficiency from 0 to 100% using a two-plasmid system.

**FIG 5:**
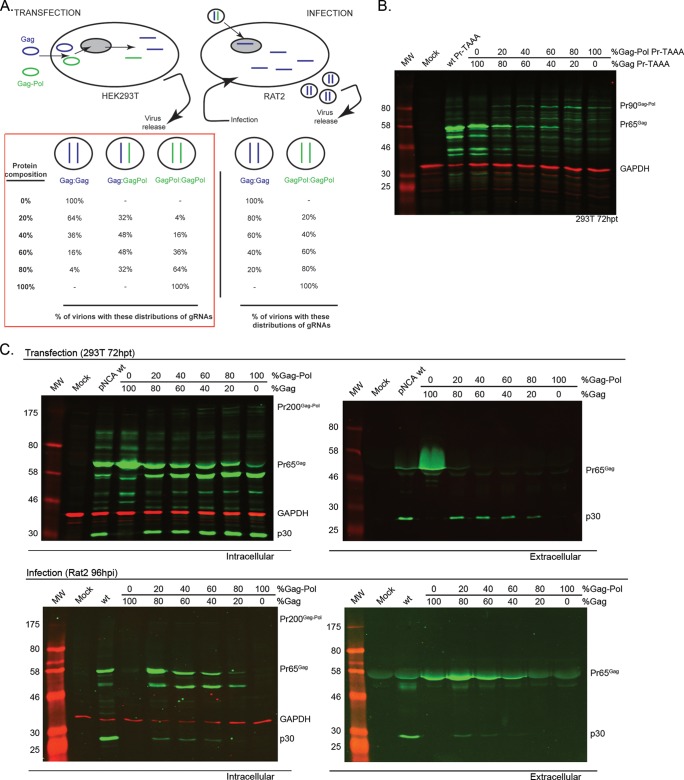
Two-plasmid system for modulating effective readthrough efficiency. (A) Schematic representation of the system. Proviral plasmids providing Gag protein only (pNCA-S1d mutant) or Gag-Pol protein only (pNCA-CAG mutant), represented as blue and green, respectively, were transfected into 293T cells. Each cell is estimated to receive an average of 500,000 copies of plasmid; thus, all cells are highly likely to take up the correct proportion of plasmids on transfection. RNAs corresponding to the plasmid sequence would be expressed and used as a template for translation of Gag/Gag-Pol and for packaging in the dimer form. Virion particles of three genotypes would be produced (boxed in red), with two homodimers and a heterodimer segregating according to the Hardy-Weinberg principle (a^2^ + 2ab + b^2^). At 72 hpt released virions were harvested and used to infect fresh Rat2 cells as previously described. One copy of the genome would integrate and therefore produce a homodimer form, either Gag or Gag-Pol expressor only (right panel). (B) 293T cells were transfected with different proportions of truncated protease-defective (Pr− TAAA) (see the text) variants of S1d and CAG proviral plasmids to confirm increasing Gag-Pol synthesis with increasing ratio of transfected Gag-Pol/Gag. At 72 hpt cell lysates were analyzed by 10% SDS-PAGE and immunoblotting using a polyclonal anti-p30 serum. GAPDH was used as a loading control. Gag (Pr65^Gag^) and truncated Gag-Pol (Pr90^Gag-Pol^) products are labeled. (C) 293T cells were transfected with different proportions of pNCA-S1d (Gag only) and/or pNCA-CAG (Gag-Pol only) plasmid providing an effective readthrough efficiency from 0 to 100% (upper panel), and released virus was used subsequently to infect Rat2 cells (lower panel). Cell lysates and supernatant virus were analyzed as described earlier. In panels B and C, molecular weights (MW; in thousands) are indicated to the left of the blots.

To investigate a broader range of RTEs (from 0 to 100%), we employed a two-plasmid system approach similar to that of Shehu-Xhilaga et al. (14). Proviral plasmids providing almost exclusively Gag (pNCA-S1d; residual RTE of 0.21%) or Gag-Pol only (pNCA-CAG mutant, in which a glutamine codon replaced the original stop codon to ensure an RTE of 100%) were transfected into 293T cells in proportions to yield effective RTEs of 0, 20, 40, 60, 80, and 100% (Fig. 5A). Here, the genomic RNAs (gRNAs) produced from episomal pNCA-S1d and pNCA-CAG and the encoded Gag and Gag-Pol polyproteins would be expressed at levels determined by the input ratio of the two plasmid DNAs. According to the Hardy-Weinberg principle, particles would segregate into three gRNA combinations: homodimeric for S1d or CAG alone or heterodimeric genotypes for mixtures (Fig. 5A, boxed in red). Following infection of Rat2 cells, each infecting virion would, in principle, integrate only one copy of the viral genome and would therefore produce either Gag or Gag-Pol polyproteins, but not both. Thus, virions released from Rat2 cells would be noninfectious, and this experimental system permits only a single round of infection to be studied. We began by confirming that the two-plasmid system generated the expected continuum of readthrough efficiencies. To facilitate this, we prepared variants of pNCA-S1d and -CAG with a premature stop codon in the viral protease (termed Pr− TAAA) to inactivate the enzyme and to truncate the size of the Gag-Pol polyprotein to improve detection by immunoblotting. These constructs were transfected into 293T cells in different proportions, and at 72 hpt, cells were harvested. Immunoblotting of infected cells (Fig. 5B) confirmed an increase in levels of the truncated Gag-Pol (Pr90^Gag-Pol^) in comparison to Gag (Pr65^Gag^), concomitant with the increase in predicted RTE. The minor products of proteolysis seen in this experiment presumably arise from the action of a cellular protease(s).

**FIG 6:**
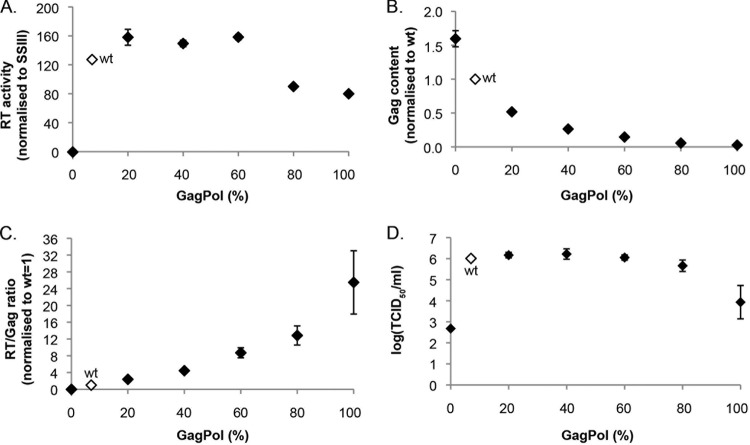
Characteristics of virion particles produced from the two-plasmid system. 293T cells were transfected with different proportions of pNCA-S1d (Gag only) and/or pNCA-CAG (Gag-Pol only) plasmid providing an effective readthrough efficiency from 0 to 100%. (A) RT assays of viral supernatants, quantified in triplicate on a Typhoon scanner. Values represent the means of two independent experiments with standard errors. RT activity was normalized to a defined activity of SuperScript III reverse transcriptase (Invitrogen/Life Technologies) (1 RT unit is equivalent to 1 μU of SuperScript III). (B) Two-fold serial dilutions of viral supernatants were subjected to dot blot analysis using the anti-p30 antibody, and Gag content was quantified using a Li-Cor scanner. Values represent the means of two independent experiments with standard errors. (C) The RT/Gag ratio was calculated for each sample and is shown normalized to the WT value, set as 1. Error bars represent standard errors. (D) TCID_50_ assays were performed with supernatant serial dilutions as previously described. Values denote the means of duplicate titrations from two independent experiments, each internally normalized to the WT titer. Error bars represent standard errors. Note that the 0% RTE virus derived from mutant pNCA-S1d retains a low level of Gag-Pol expression (Fig. 2A and C), which likely accounts for the fact that some replicative activity was retained.

Subsequently, pNCA-S1d and pNCA-CAG were transfected in 293T cells in the proportions that would deliver RTEs of 0 to 100%, and at 72 hpt, cells were harvested, lysed, and subjected to immunoblotting (Fig. 5C, upper panels). As the effective RTE increased, processed forms of Gag and Gag-Pol were more evident, with the unprocessed Gag form (Pr65^Gag^) almost absent at 100% RTE. Consistent with this, at 80% and 100% readthrough, viral particle production was noticeably diminished. It is highly likely that overproduction of the full-length Gag-Pol polyprotein increased intracellular protease activity to a level detrimental for viral replication; similar observations have been made for HIV-1 (19, 20). In contrast, at 0% RTE, the only protein observed in the extracellular medium was unprocessed Gag, which accumulated to high levels. As mentioned earlier, these particles are likely to be immature due to the lack of viral protease. Filtered supernatant from transfected cells was then used to infect subconfluent Rat2 cells. Intra- and extracellular protein analysis by immunoblotting (Fig. 5C, bottom panels) revealed that productive infection was achieved in the intermediate RTE range (20 to 60%) but not at the extremes (0%, 80%, and 100%). To assess virion composition in these experiments, we performed RT assays and quantified Gag by dot blotting. RT activities increased up to 60% RTE but dropped dramatically at 80 and 100% RTE (Fig. 6A), almost certainly due to the lower levels of particle production, as observed by immunoblotting (Fig. 5C). Quantitative dot blots (Fig. 6B) revealed a 33-fold reduction in Gag levels between WT and 100% RTE viruses. As shown in Fig. 6C, the RT/Gag ratio observed for the various RTEs (normalized to the WT virus) showed a positive linear correlation (*R*^2^ = 0.89), suggesting that the assembly process in MuLV does not preclude incorporation of excess Gag-Pol. TCID_50_ assays performed using this two-plasmid system (Fig. 6D) revealed that virus infectivity was essentially unaltered between 20 and 60% RTE and was almost identical to that of the WT virus. Even at 80% RTE, the TCID_50_ titer decreased by only 2.2-fold. Testing the 0 and 100% RTE viruses separately confirmed that neither virus was able to replicate well, with viral titers reduced 100- to 1,000-fold.

### Analysis of virion RNA in particles generated in the two-plasmid system.

To confirm that the viral RNA released from 293T-transfected and Rat2-infected cells in the two-plasmid experiment corresponded to the ratios expected and that no bias was established during packaging, virion RNA was extracted and sequenced. PCR amplicons (∼550 bp) covering the readthrough signal and flanking regions were prepared and sequenced, and these data are summarized in Table 1. The RNA composition of virions produced from 293T-transfected cells very accurately reflected the transfected DNA sequences. WT transfection produced exclusively WT sequences, and the same was true for S1d (0%) and CAG (100%) transfections. Importantly, different RTE combinations showed no bias and largely retained the input ratios. A small proportion of the sequenced readthrough signals had accumulated point mutations, mostly within loop 3, but the relative proportions of the input vector remained as expected. Following infection, the genomic content of released virions was dramatically changed. At the proportions where we could recover sufficient amplicon to generate sequences from several (∼40) virions (20, 40, and 60%), we saw almost exclusively the S1d vector genome. At 80% RTE, very few sequences (six) were obtained, and of these, one-third were S1d, and the other two-thirds were revertants (CAG to UAG WT). No sequence information could be recovered from the 100% CAG infections. These data support the view that the CAG clone is noninfectious, yet the S1d virus retains a low level of infectivity.

**TABLE 1:** RNA sequence composition of particles released from the two-plasmid system

Cell line and Gag-Pol proportion (%)^*a*^	% RNA sequence identity by strain^*b*^			
	Wild type	S1d mutant	CAG mutant	Other
293T				
Wild type	100	0	0	0
0	0	100 (5.00)	0	0
20	0	81.25 (3.12)	18.75	0
40	0	77.77 (16.6)	22.23	0
60	0	47.36 (5.26)	47.36	5.28
80	0	52.63	47.37	0
100	0	0	100 (5.55)	0
Rat2				
Wild type	100	0	0	0
0	0	100 (11.76)	0	0
20	0	100 (8.33)	0	0
40	0	92.30	0	7.70
60	0	91.66 (16.66)	8.34	0
80	66.66	33.34 (11.11)	0	0

a 293T cells were transfected with different proportions of pNCA-S1d (Gag only, represented by 0% Gag-Pol) and/or pNCA-CAG (Gag-Pol only) providing an effective readthrough efficiency from 0 to 100%, and released virus was used subsequently to infect Rat2 cells. The culture medium was sterile filtered, and viral RNA was isolated and reverse transcribed using avian myeloblastosis virus RT. The resulting cDNA was amplified by PCR. PCR amplification products were cloned into the pGEM-T-Easy vector (Promega), and different clones were sequenced from 293T-transfected cells and Rat2-infected cells.

b The numbers in parentheses show the percentage of the respective sample that contains one or more point mutations.

### Validation of the two-plasmid system at lower RTEs.

**FIG 7:**
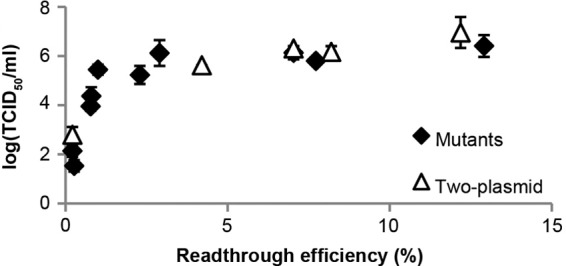
Validation of the two-plasmid system. 293T cells were transfected with different proportions of pNCA-S1d (Gag only) and/or pNCA-CAG (Gag-Pol only) plasmid to generate an effective readthrough efficiency from 0 to 12%, and released virus was used subsequently to infect Rat2 cells. TCID_50_ assays were performed with supernatant serial dilutions as described above. Values denote the means of duplicate titrations from two independent experiments Error bars represent standard errors. For comparison, these data are shown alongside those derived from the individual virus mutants (Fig. 2C).

To further validate the two-plasmid system, 293T cells were transfected with combinations that would yield a lower range of effective RTEs (0, 4, 8, and 12%), and virus replication was assayed. It was found that the TCID_50_ titers of viruses derived using the two-plasmid system could essentially be superimposed on those of individual viruses containing *cis*-acting mutations within the readthrough signal (Fig. 7). Thus, the two-plasmid system faithfully mirrors the infectivity of individual viruses with various readthrough efficiencies.

## DISCUSSION

Translational recoding signals are potentially exploitable as targets for antiviral intervention (16, 49, 50), and supporting this, replication of the Saccharomyces cerevisiae L-A totivirus (50–52), HIV-1 (reviewed in reference 16), and the severe acute respiratory syndrome (SARS)-associated coronavirus (53) has been shown to be sensitive to quite subtle changes in ribosomal frameshifting frequency. Here, we describe the first systematic study of the effect of modulating programmed stop codon readthrough efficiency on virus replication, using MuLV as a model system. Reductions in readthrough led to a substantial replication defect or abolished replication, but a 2-fold stimulation had little effect, and surprisingly, up to an ∼8.5-fold stimulation of readthrough (equivalent to up to 60% readthrough) was well tolerated. Such tolerance to increased recoding frequency may also be the case for another simple retrovirus, RSV (24).

That a decrease in readthrough is detrimental to MuLV replication is consistent with other studies. Although reducing Gag-Pol levels does not prevent particle assembly and release (7, 54), it decreases incorporation of essential virus enzymes into virions, with consequent effects on subsequent rounds of infection, as has also been observed for RSV (24) and HIV-1 (28, 29, 55). Indeed, we noted a linear relationship between RTE and virion RT/Gag composition, and this correlated closely with infectivity at RTEs below the WT value. At very low RTEs (S1d and SP2), we observed high levels of unprocessed Gag in the extracellular medium from transfected 293T cells, supporting previous observations that Gag-Pol has an inhibitory effect on particle production (14, 19, 20, 23). The design of synonymous readthrough-inhibitory mutations was relatively straightforward, thanks to the substantial literature on the MuLV pseudoknot structure (6, 8–10, 56). However, it proved more challenging to stimulate readthrough frequency. We were able to identify a synonymous stem-stabilizing mutation (S1GC) of the MuLV readthrough pseudoknot that increased readthrough some 1.5-fold, but this had no effect on replication. Subsequently, additional nonsynonymous mutations were introduced into the pseudoknot, designed based on recent NMR data (10), that led to a greater stimulation of readthrough (to some 2.5-fold above the WT signal). Unfortunately, however, these mutants could not be used in the assessment of virus fitness as all resulted in a defective protease and failed viral polyprotein processing. The key mutation affected a protease Ile residue which, in comparison with the structures of the HIV-1 (57) and xenotropic murine leukemia virus-related virus (XMRV) proteases (58), is located in the β1 strand forming the dimer interface. Amino acid substitutions in this region may thus have altered the interaction with the other monomer, rendering the protease dysfunctional.

Given the limited scope for introducing stimulatory, nonsynonymous changes within the readthrough signal, we also tested two strategies to increase RTE generally, namely, depletion of release factors and aminoglycoside supplementation. In Rat2 cells depleted of eRF1 or eRF3, virus replication was uniformly poor, presumably a consequence of the global translation termination defect, which made any specific effects of release factor depletion on replication difficult to discern. In a separate experiment, addition of the readthrough-stimulatory aminoglycoside gentamicin reproducibly stimulated MuLV readthrough some 1.5- to 2.0-fold but, again, without affecting replication. It should be noted that many previous studies have looked at the effect of aminoglycosides on readthrough of premature termination codons (PTCs) but not of programmed readthrough sites. In contrast to what has been observed with PTCs, gentamicin did not greatly stimulate MuLV readthrough, perhaps because this site already has a high basal RTE or because there is a limit (20%) to gentamicin action. Interestingly, the effects of gentamicin and stimulatory pseudoknot mutations (S1GC and NMR series) were synergistic, similar to what has been observed in studies on the stimulatory effects of polyamines or rpL4 on MuLV readthrough (12, 59). Mechanistically, this could reflect differential but complementary interactions with the ribosome or modulations of the ribosomal complex to induce readthrough. Equally, however, the limits observed may suggest that the pseudoknot induces changes to the decoding site similar to those brought about by aminoglycosides (60).

As an alternative means to facilitate investigation of a broader range of RTE stimulation, we utilized a two-plasmid approach pioneered by Shehu-Xhilaga and colleagues (14) in their studies of HIV-1 replication and frameshifting. This approach allowed us to generate a range of RTEs (from 0 to 100%) and, at the upper end, very high Gag-Pol/Gag ratios in transfected cells. This single-cycle assay system is expected to produce virions with gRNAs in ratios that reflect the transfection ratios. A concern was the potential differential sensitivity of the two gRNA transcripts to degradation via the nonsense-mediated mRNA decay pathway (61). However, immunoblotting of cells transfected with 0%, 100%, and intermediate effective RTEs showed no obvious differences in overall Gag translation, arguing against any differential degradation of one or other of the two gRNA species. To validate the two-plasmid system, we used pNCA-S1d and -CAG to generate a lower RTE gradient, from 0 to 12%. The titers from these transfections were very similar to those obtained from individual viruses with *cis*-acting mutations within the pseudoknot.

The large increases in intracellular Gag-Pol levels at high RTEs had a number of striking effects on the viruses produced. The particle number dropped ∼30-fold between the WT level and 100% RTE, possibly due to premature protease activation triggered by higher Gag-Pol concentrations, supporting previous studies that show an inhibitory effect of Gag-Pol on particle production (14, 19, 20, 23). As for HIV-1 and RSV (24, 29, 55, 62), we found that virion composition was highly susceptible to changes in RTE. Indeed, the RT/p30 ratio increased in consonance with a higher RTE, as observed previously for RSV (up to 3-fold increase tested) (24). This supports the idea that virion composition in MuLV is specified at the level of translation. Our analysis also revealed that in the two-plasmid system, the gRNA is packaged at ratios consistent with the transfection ratio, arguing against any packaging biases. In subsequent infections, however, a wide variety of changes were observed. The S1d species was predominant, almost certainly due to the fact that in virus assays it exhibits higher titers and higher numbers of particles (∼50-fold higher p30 levels). At 80% RTE, about half of the recovered sequences were WT, which may have been the result of a single misincorporation error in reverse transcription (CAG to UAG) or strand switching between heterodimeric genomes. In either case, the resultant WT genomic RNA would be expected to outcompete CAG genomic RNA in particle production.

Surprisingly, as revealed in TCID_50_ assays, virus infectivity was essentially unaltered between 20 and 60% RTE and was almost identical to that of the WT. At 80% RTE, the titer measured decreased by only 2.2-fold. Examination of 0 and 100% RTE viruses separately showed that neither virus was able to replicate well, with titers reduced by 100- to 1,000-fold. Thus, an ∼8.5-fold stimulation of RTE was tolerated before viral replication was compromised, a value far higher than has been shown for other retroviruses (14, 24). How can this be accounted for? One possibility is that in these tissue culture systems, the WT Gag-Pol/Gag ratio is not, in fact, optimal. The substantial quantities of immature Gag seen in extracellular WT virus (and reported in other studies [7, 12]) may indicate that not all particles have undergone maturation. Further, Gag-Pol may ameliorate this and increase specific infectivity. The maintenance of infectivity at higher Gag-Pol levels could represent a positive effect of extra Gag-Pol on infectivity, balancing a negative effect on virion assembly. The question remains as to why the virus naturally maintains a Gag-Pol/Gag ratio of ∼5%. It may be that this is a constraint imposed by coding restrictions, but more probable is that in the natural host, constraints posed by the immune system have effects we are unable to analyze in culture. The window of effective readthrough may also be narrower as even minor replication defects could substantially reduce fitness. A limitation of the present work is that higher RTEs could be achieved only through the use of the two-plasmid system which precludes the investigation of subtle effects on fitness in multiple-passage experiments.

Readthrough is employed by many RNA virus families (63), and like ribosomal frameshifting (64), is a potential target for antiviral therapeutics. Pathogenic viruses utilizing readthrough include feline leukemia virus (65), Venezuelan equine encephalitis alphavirus (66), and Colorado tick fever virus (67). Taking into account the present data and earlier studies, it is clear that small-molecule inhibitors of readthrough would seem to be the favored class since even a 4-fold effect would be likely to severely compromise replication.
